# The interplay of ferroptosis and oxidative stress in the pathogenesis of aortic dissection

**DOI:** 10.3389/fphar.2025.1519273

**Published:** 2025-02-05

**Authors:** Zhaoshan Zhang, Haichao Wang, Xi Kan, Xiaozhao Zhang, Senping Xu, Jie Cai, Jiawei Guo

**Affiliations:** ^1^ Department of Vascular and Endovascular Surgery, The First Affiliated Hospital of Yangtze University, Jingzhou, China; ^2^ Department of Stomatology, The First Affiliated Hospital of Yangtze University, Yangtze University, Jingzhou, China; ^3^ Department of Pharmacology, School of Medicine, Yangtze University, Jingzhou, China

**Keywords:** aortic dissection, ferroptosis, vascular smooth muscle cells, oxidative stress, ROS, endothelial cells

## Abstract

Aortic dissection (AD) is a life-threatening vascular condition marked by the separation or tearing of the aortic media. Ferroptosis, a form of iron-dependent programmed cell death, occurs alongside lipid peroxidation and the accumulation of reactive oxygen species (ROS). The relationship between ferroptosis and AD lies in its damaging effect on vascular cells. In AD, ferroptosis worsens the damage to vascular smooth muscle cells (VSMCs) and endothelial cells (ECs), thereby weakening the vascular wall’s structural integrity and accelerating the onset and progression of the condition. However, the molecular mechanisms through which ferroptosis regulates the onset and progression of AD remain poorly understood. This article explores the relationship between ferroptosis and AD.

## 1 Introduction

AD is an age-associated, life-threatening cardiovascular disorder that often results in fatal outcomes, with many patients dying before hospital admission or a definitive diagnosis is established. The Stanford classification of AD, introduced in 1970, continues to be widely used today (Daily et al., 1970). According to the widely recognized Stanford classification, ADs are categorized into Type A and Type B. Type A dissections are the more severe form and typically require urgent surgical intervention for recovery. In contrast, Type B dissections exhibit a lower likelihood of acute complications compared to Type A; nonetheless, they still pose a significant risk to life (Peterss et al., 2016). The pathogenesis of AD arises from a multifactorial interplay, involving abnormalities in the structure of the vascular wall, hypertension, inflammation, oxidative stress, apoptosis, ferroptosis, and various genetic mutations (Vilacosta et al., 2010; Gawinecka et al., 2017). AD can lead to a multitude of complications (Gawinecka et al., 2017),including aortic rupture, cardiogenic sudden death, visceral ischemia, ischemic stroke, subarachnoid hemorrhage, renal failure, and heart failure (Grond-Ginsbach et al., 2010). Prompt recognition and management of these complications are crucial to improving the survival rate of patients with AD.

**FIGURE 1:**
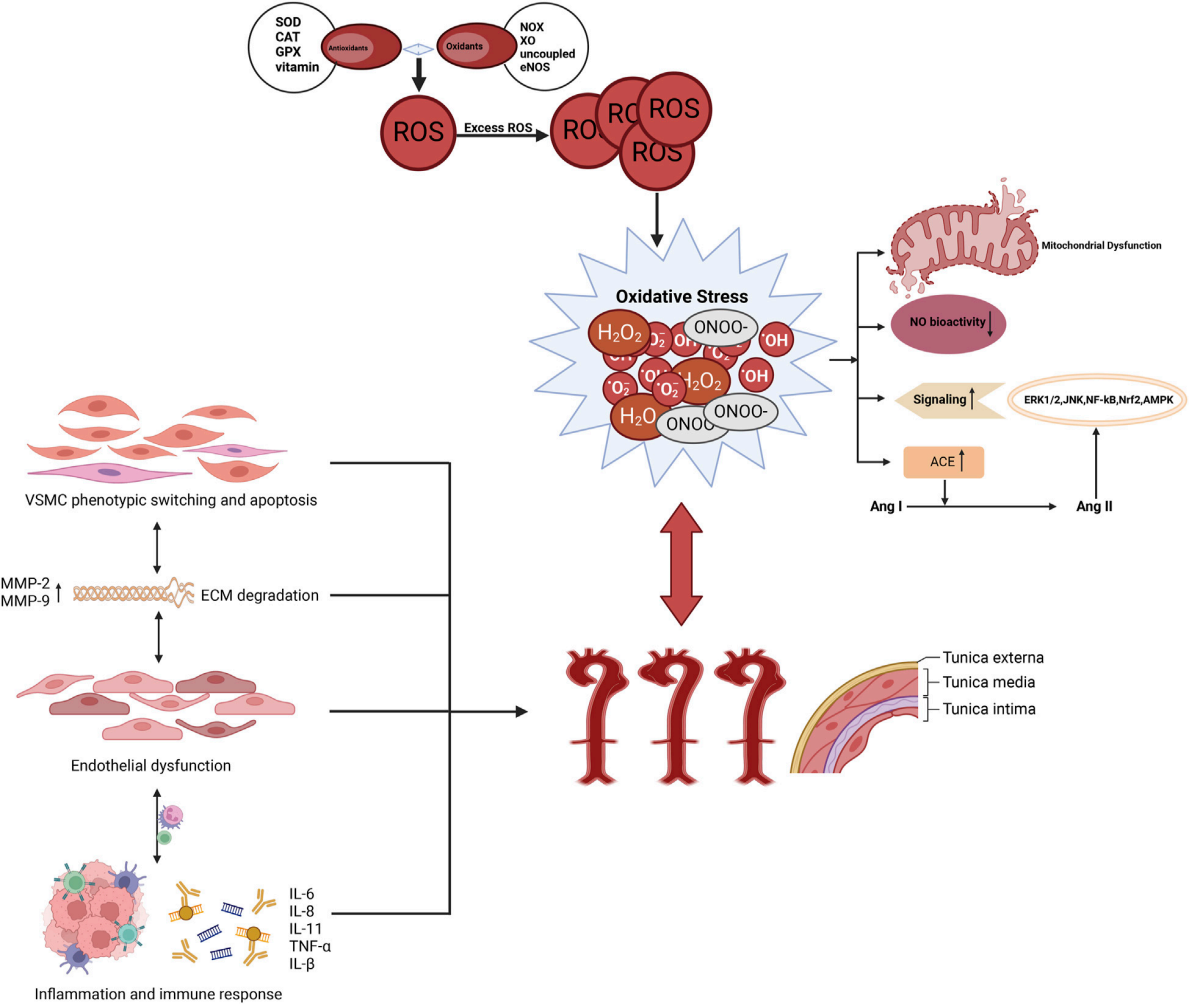
The association between AD and oxidative stress. Oxidative stress increases the production of ROS, which promotes apoptosis and pathological phenotypic transformation of vascular smooth muscle cells in the aortic media. It also induces the overexpression of proteolytic enzymes, such as MMPs, leading to ECM degradation. Furthermore, oxidative stress triggers inflammatory responses, attracting the infiltration of macrophages and mononuclear lymphocytes, thereby further compromising the structural integrity of the aortic wall and accelerating the formation of AD.

The development of cardiovascular diseases is intricately linked to various regulated forms of cell death, including apoptosis, ischemic necrosis, pyroptosis, and autophagy (Del Re et al., 2019). Morphologically, biochemically, and genetically, ferroptosis differs from apoptosis, autophagy, and other forms of necrosis. It primarily involves the accumulation of lethal lipid peroxides catalyzed by iron, leading to oxidative damage to the cell membrane (Figure 1) (Stockwell et al., 2017). Both primary and secondary iron overload, as well as iron deficiency, exert significant effects on the heart (Jankowska et al., 2014; Berdoukas et al., 2015). Ferroptosis plays a pivotal role in cardiomyopathy, ischemia-reperfusion injury (IRI), myocardial infarction (MI), and heart failure (HF), and is recognized as a fundamental mechanism underlying numerous cardiovascular diseases (Figure 1). In the aortas of patients with AD, the levels of iron-death-associated molecules, such as transferrin receptor (TFR), Heme oxygenase-1 (HO-1), and ferritin, were increased, while the levels of iron-death-regulating proteins, including SLC7A11, GPX4, and FSP1, were decreased (Figure 1) (He X. et al., 2024).

This article aims to explore the potential connections and research significance of AD and ferroptosis by systematically reviewing the pathophysiological mechanisms of AD, as well as the characteristics of ferroptosis and its role in cardiovascular diseases.

## 2 The mechanistic link between ferroptosis and AD

**FIGURE 2:**
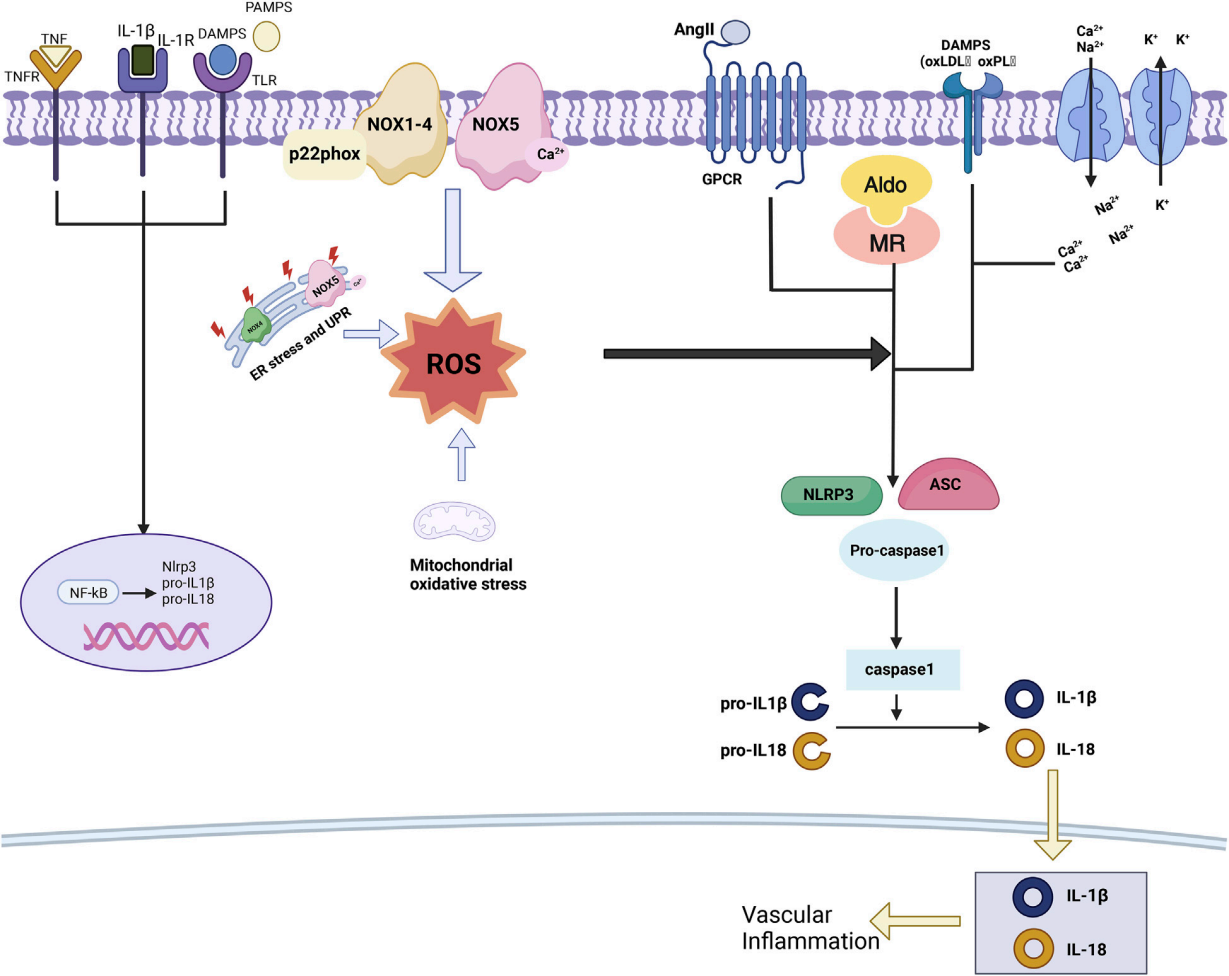
Oxidative stress in hypertension. ROS act as mediators in the activation of the NLRP3 inflammasome by hypertensive stimuli, prompting the secretion of pro-inflammatory cytokines, as well as pathogen-associated molecular patterns (PAMPs) and danger-associated molecular patterns (DAMPs). This sequence of events triggers the activation of NF-κB and the expression of inflammasome components. Furthermore, ROS play a pivotal role in regulating the formation of the inflammasome complex, which consists of NLRP3, ASC, and pro-caspase-1, through their engagement with PAMPs, DAMPs, oxidized low-density lipoprotein (oxLDL), oxidized phospholipids (oxPL), AngII, aldosterone, and cations. Ultimately, this mechanism culminates in the activation of caspase-1, resulting in the proteolysis of pro-IL-1β and pro-IL-18.

Heightened oxidative stress in AD induces ferroptosis-related lipid peroxidation reactions. Oxidative stress plays a crucial role in the pathogenesis and progression of AD and is closely linked to ferroptosis, a form of cell death (Figure 2). The following section explores the potential mechanisms by which oxidative stress is elevated in AD, leading to ferroptosis-related lipid peroxidation.

### 2.1 Oxidative stress and AD

The pathophysiology of AD is driven by multiple factors centered around the disruption of aortic wall integrity. This disruption may arise from congenital structural instability of the aortic wall, often due to inherited connective tissue disorders, or from acquired factors like age-related atherosclerotic degeneration (Nienaber et al., 2016). The aorta consists of three primary layers: the intima, the media, and the adventitia. VSMCs within the media play a crucial role in maintaining the structural integrity of the aorta (Chen et al., 2019). The primary histological abnormalities linked to AD include degenerative lesions of the intima-media layer, disruption of elastic fibers, apoptosis, reduced smooth muscle cell count, extracellular matrix degradation, and inflammatory cell infiltration. These abnormalities interact and collectively increase the vulnerability of the aortic wall, precipitating the onset and progression of AD (Bonderman et al., 1999).

Clinically, AD is classified based on the location and extent of aortic involvement, providing essential guidance for diagnosis and treatment (Shin et al., 2024). The Stanford classification categorizes AD into Type A, involving the ascending aorta, and Type B, restricted to the descending aorta (Varela-Jaramillo et al., 2024). Alternatively, the DeBakey classification offers a more detailed anatomical framework: Type I involves both ascending and descending aorta, Type II is limited to the ascending aorta, and Type III originates in the descending aorta, with subtypes IIIa (thoracic descending aorta) and IIIb (extending into the abdominal aorta) (MacGillivray et al., 2022).

**FIGURE 3:**
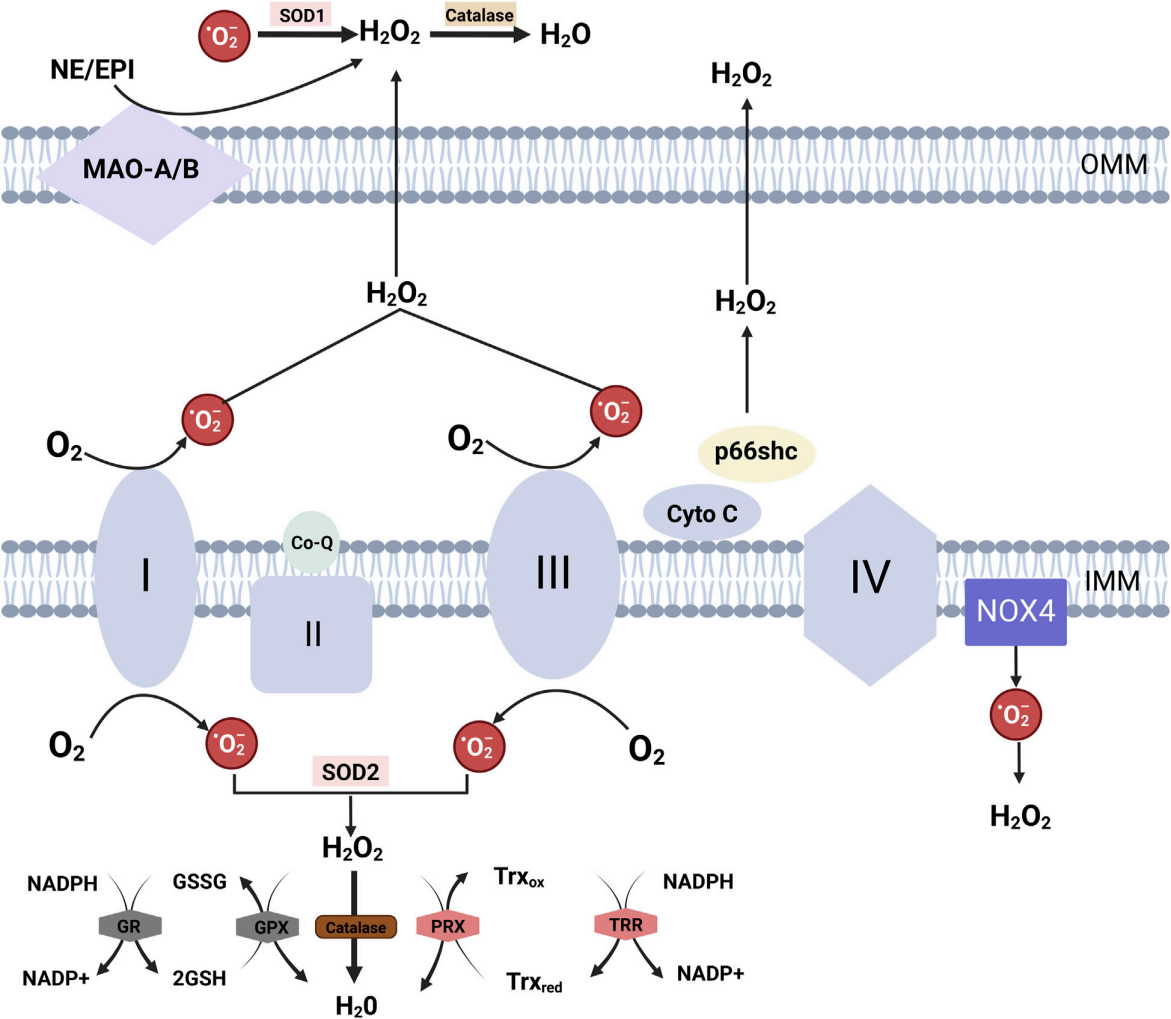
Mitochondrial ROS Generation and Detoxification Mechanisms Electron transfer at complexes I and III of the respiratory chain results in the production of superoxide anions (O₂⁻). During electron transfer from complex III to complex IV via cytochrome C, p66Shc can intercept electrons from cytochrome C, derived from complex III, leading to cytochrome C oxidation and the generation of O₂⁻. These superoxide anions are subsequently converted into H₂O₂ by SOD. NOX4, localized to the inner mitochondrial membrane, produces both O₂⁻ and H₂O₂ within the mitochondrial matrix. Monoamine oxidase isoforms A and B (MAO-A/B), situated in the mitochondrial outer membrane, degrade monoamines into aldehydes and H₂O₂. Mitochondrial H₂O₂ detoxification is catalyzed by glutathione peroxidases (Gpx1 and Gpx4) and peroxiredoxins (PRX3 and PRX5) within mitochondria. These enzymes oxidize glutathione (GSH) to glutathione disulfide (GSSG), which is then reduced back to GSH by glutathione reductase (GR), utilizing NADPH as the reducing equivalent. Additionally, PRX oxidizes thioredoxin (Trx), particularly the mitochondrial isoform Trx2, and the reduced thioredoxin pool is regenerated by NADPH-dependent thioredoxin reductase (TRR2) in the mitochondrial matrix.

Oxidative stress has been shown to promote pathological phenotypic transformation and apoptosis of smooth muscle cells, increase the expression of protein hydrolases such as matrix metalloproteinases (MMPs), induce extracellular matrix (ECM) degradation, and stimulate fibroblast proliferation and infiltration of macrophages and mononuclear lymphocytes (Figure 3). This process disrupts the structure and function of ECs, ultimately leading to endothelial dysfunction, which contributes to pathological remodeling of the aortic wall and drives the development of AD (Branchetti et al., 2013; Higashi, 2022). Further exploration of the potential value of oxidative stress in the diagnosis, prognostic assessment, and treatment of AD could be facilitated by a more detailed investigation of its role in this process (Figure 3).

#### 2.1.1 The sources of oxidative stress in AD

The primary sources of oxidative stress in aortic dissection (AD) include the overproduction of ROS from NADPH oxidase (NOX) activation, mitochondrial dysfunction, and oxidized lipids. Inflammatory cells, such as neutrophils and macrophages, further amplify ROS production, disrupting redox balance and promoting vascular injury in AD.

##### 2.1.1.1 Oxidative stress in hypertensive states

Hypertension is a major contributor to AD, as it induces VSMC dedifferentiation, which may lead to the formation of aortic aneurysms and dissections (Zhang et al., 2017). Hypertension-induced vascular lesions include impaired endothelium-dependent diastolic function, increased arterial stiffness, enhanced contractility, inflammation, vascular calcification, and remodeling (Dikalov et al., 2014). Oxidative stress levels in the vascular wall are markedly elevated in hypertensive states. Numerous pro-hypertensive factors actively stimulate and escalate ROS production in ECs, smooth muscle cells, the adventitia, and perivascular adipose tissue, ultimately leading to vascular damage.

###### 2.1.1.1.1 NOX-mediated oxidative stress in hypertensive states

**FIGURE 4:**
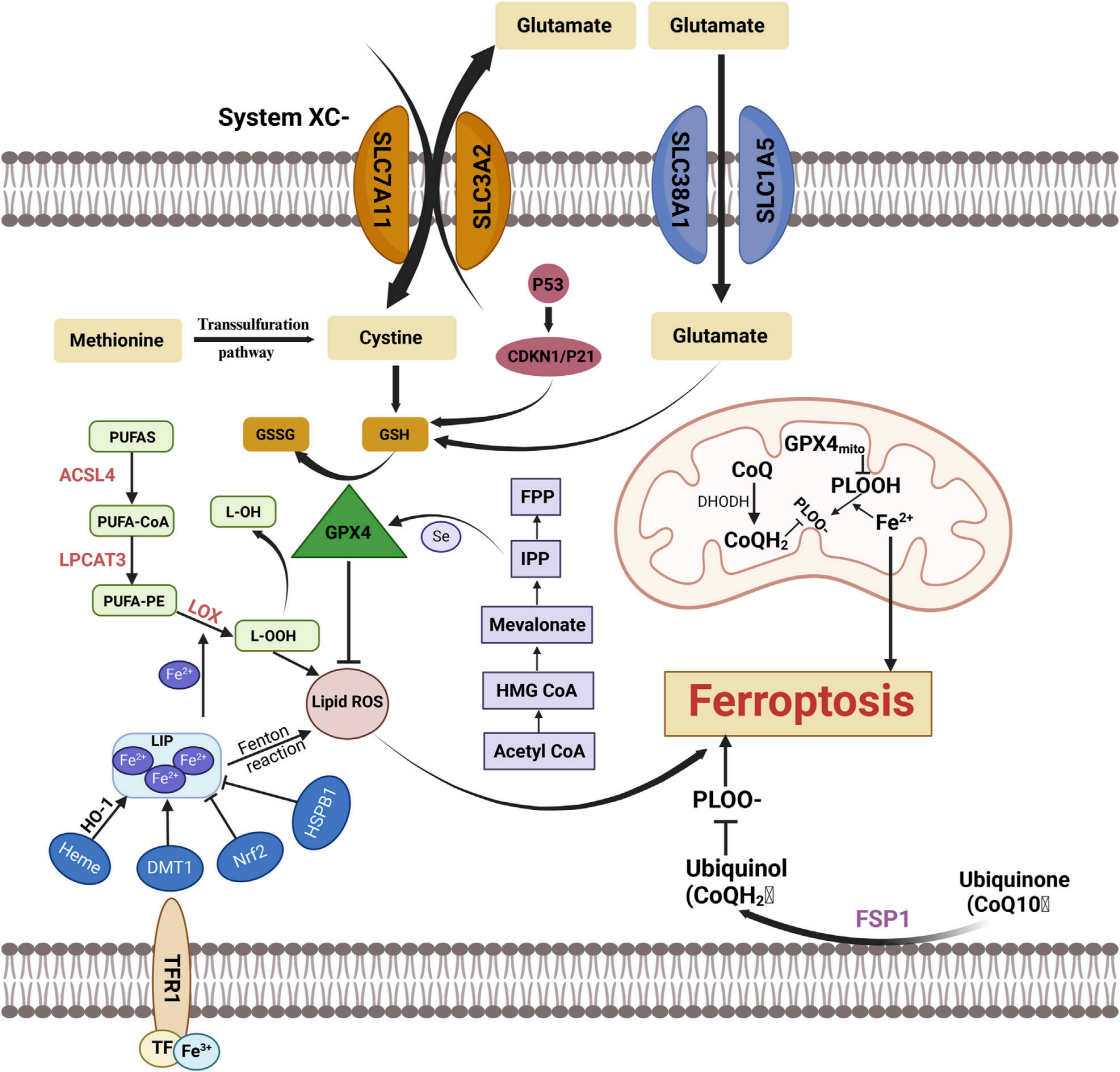
Key Mechanisms and Molecules in Ferroptosis Ferroptosis is a regulated form of cell death characterized by the accumulation of lipid peroxides and depletion of GSH, ultimately leading to cell demise. This form of cell death is distinct from apoptosis and necrosis, primarily driven by iron overload, which facilitates the generation of ROS through Fenton reactions. FPP, Farnesyl pyrophosphate; Mevalonate:Mevalonic Acid; HMG CoA, 3-Hydroxy-3-Methylglutaryl Coenzyme A; Acetyl CoA, Acetyl Coenzyme A; PUFAs, Polyunsaturated Fatty Acids; GSSG:Oxidized Glutathione; GSH, Glutathione; ACSL4, Acyl-CoA Synthetase Long-Chain Family Member 4; L-OH, Lipid Hydroxide; GPX4, Glutathione Peroxidase 4; LPCAT3, Lysophosphatidylcholine Acyltransferase 3; PUFA-PE, Polyunsaturated Fatty Acyl-Phosphatidylethanolamine; LOX, Lipoxygenase; L-OOH, Lipid Hydroperoxide; PUFA-CoA, Polyunsaturated Fatty Acyl-CoA; Lipid ROS, Lipid Reactive Oxygen Species; CDKN1/P21:Cyclin-Dependent Kinase Inhibitor 1A/P21; System Xc⁻, Cystine/Glutamate Antiporter; FPP, Farnesyl Pyrophosphate; HSPB1, Heat Shock Protein Beta-1; SLC38AT, SLC38A Transporter; GPX4mit, Glutathione Peroxidase 4 (Mitochondrial); DMT1, Divalent Metal Transporter 1; CoO:Cobalt Oxide; PLOOH, Phospholipid Hydroperoxide; PLO, Phospholipid Oxide; DHODH, Dihydroorotate Dehydrogenas; NrF2, Nuclear Factor Erythroid 2-Related Factor 2.

Non-phagocytic Nox is the primary enzymatic source of cardiovascular ROS, and Nox-derived ROS are associated with increased blood pressure in all prevalent models of hypertension (Sedeek et al., 2009; Karbach et al., 2014). The Nox family consists of seven distinct isoforms: Nox1 through Nox5, as well as Duox1 and Duox2, each playing a unique role in regulating ROS production and contributing to various physiological and pathological processes, including hypertension and vascular dysfunction (Figure 4). The activation of Nox1 through Nox4 requires the involvement of p22phox and their respective subunits. Unlike other Nox isoforms, the activation of Nox5 is independent of p22phox and the NOX subunits (Gabig and Babior, 1979). Nox1, Nox2, Nox4, and Nox5 of the Nox family are associated with oxidative stress in hypertension (Figure 2). Treatment with inhibitors targeting Nox1, Nox2, and Nox4 (such as apocynin, diphenyl iodide, gp91ds-tat, and GKT137831) in a mouse model of hypertension significantly improved vascular function, normalized blood pressure, and attenuated hypertension-induced cardiac remodeling (Schröder, 2014). This discovery strengthens the significant correlation observed among Nox activation, oxidative stress, and hypertension, highlighting the intricate interplay among these factors (Li L. et al., 2018).

###### 2.1.1.1.2 Angiotensin (Ang) II-mediated oxidative stress in hypertensive states

AngII was the first vasoactive compound identified to trigger the generation of ROS in vascular cells and the aortas of hypertensive mice under *in vitro* conditions (Griendling et al., 1994). The administration of AngII in mice, along with the examination of its impact on resistance arteries in hypertensive individuals, demonstrated a notable upregulation in the expression of Nox1 and Nox2 within the vasculature. This finding was further correlated with an increase in ROS production in both scenarios (Martínez-Revelles et al., 2017). The activation of MAP kinases, Src kinases, Rho kinases, and Ca^2^⁺ channels initiates a complex sequence of signaling events resulting from this alteration. This, in turn, leads to endothelial dysfunction, a hyperreactive state of the vasculature, vascular remodeling, infiltration of inflammatory cells into the vessel wall, increased vessel stiffness, and the development of fibrosis.

###### 2.1.1.1.3 Inflammatory vesicle-mediated oxidative stress in hypertensive states

The NLRP3 inflammasome, a member of the NOD-like receptor family containing a pyrin domain, exerts a prominent effect in vascular inflammatory processes, playing a crucial role in hypertension-related vascular inflammation (Pasqua et al., 2018). The NLRP3 inflammasome is a complex multiprotein assembly, with core components including ASC (apoptosis-associated speck-like protein), an articulating molecule, and procaspase-1, a precursor protease. When exposed to stimuli such as immune activators, cellular stress, or oxidative stress, the molecular conformation of NLRP3 undergoes a transformation. This alteration facilitates the interaction between NLRP3, ASC, and procaspase-1, leading to the formation of an active NLRP3 inflammasome. Once assembled, the NLRP3 inflammasome induces the autocleavage of procaspase-1, converting it into the active form of caspase-1. The activated caspase-1 triggers pyroptosis and cleaves and activates two significant pro-inflammatory cytokines: IL-1β and IL-18. The release of these cytokines amplifies and activates the body’s pro-inflammatory signaling pathways, eliciting a more extensive inflammatory response (Harijith et al., 2014). The activation of the NLRP3 inflammasome plays an instrumental role in promoting the phenotypic transformation of VSMCs induced by AngII, as well as driving cellular proliferation, vascular remodeling, and the progression of hypertension (Sun et al., 2017).

##### 2.1.1.2 Oxidative stress in mitochondrial dysfunction

The processes of the tricarboxylic acid cycle (TCA) and oxidative phosphorylation (OXPHOS) are crucial for mitochondrial ATP production, which is vital for sustaining normal physiological functions of arteries and modulating the quiescent contractile state of VSMCs (Figure 4) (Zhang and Gao, 2021). The mitochondrial respiratory chain is the primary source of intracellular ROS and plays a central role in energy production. Mitochondrial dysfunction also emerges as a novel mechanism influencing the phenotypic transformation of VSMCs and playing a pivotal role in regulating arterial remodeling.

The respiratory chain primarily generates ROS from Complex I (NADH: ubiquinone oxidoreductase) and Complex III (ubiquinol: cytochrome c oxidoreductase) (Figure 3) (Murphy et al., 2016). Normally, ATP is produced through the establishment of an electrochemical gradient by electron transfer between electron transport chains (ETC.), which regulates K+ channels and ensures proper organelle performance and cellular energy balance (Itani et al., 2016). However, under pathological conditions, mitochondrial function is compromised, resulting in increased electron leakage and subsequent superoxide anions (O₂⁻) production (Rubattu et al., 2014). Superoxide dismutase (SOD) catalyzes the dismutation of O₂⁻ into hydrogen peroxide (H₂O₂), which then traverses the mitochondrial membrane and infiltrates the cytosol (Rubattu et al., 2014). Within mitochondria, the generation of ROS is not limited to the, ETC., but also involves various proteins, including p66shc and NOX (Figure 3) (Giorgio et al., 2005; Bedard and Krause, 2007). Furthermore, uncoupled eNOS within mitochondria enhances the production of mitochondrial ROS (mtROS) in ECs (Chen et al., 2014). Notably, mtROS can oxidize the, ETC., resulting in functional disturbances of, ETC., complexes and a subsequent increase in ROS production (Incalza et al., 2018).

NOX activation is an early response of ECs to AngII. AngII increases ROS production in mitochondria and induces mitochondrial dysfunction via the activation of endothelial cell NOX2 through a PKC-dependent pathway (Dikalov et al., 2014). The basal generation of H₂O₂ is attributed to NOX4 (Takac et al., 2011), and knocking down NOX4 reduces the production of two ROS species: mitochondrial superoxide and hydrogen peroxide (Takac et al., 2011). NOX2 senses NOX4-derived H₂O₂, which in turn phosphorylates p66Shc at the Ser36 locus via mitochondrial PKC, promoting mtROS generation (Figure 2) (Kim et al., 2017). The activity of mitochondrial electron transport chain complex I is specifically inhibited by mitochondrial NOX4, resulting in decreased mitochondrial oxidative phosphorylation capacity and accelerated production of respiratory chain-derived ROS (Kozieł et al., 2013).

The p66shc adaptor protein, which is broadly expressed, serves as a typical scaffold protein. Beyond its role in cellular signaling, p66shc functions as an oxidoreductase that produces ROS through a mitochondria-dependent mechanism (Giorgio et al., 2005). Upon cellular stress, p66shc translocates to the mitochondria, where it promotes the accumulation of ROS, ultimately leading to caspase activation and the induction of apoptosis. In the absence of cellular stress, peroxidase 1 (Prx1) and p66shc form the p66shc-Prx1 complex, which sequesters p66shc in the cytoplasm and prevents mitochondrial translocation (Gertz et al., 2009). The mitochondrial translocation of p66shc is regulated by the PKC family, which triggers the mitochondrial translocation of p66shc by phosphorylating the Ser36 site of the p66shc CH2 structural domain (Mehta and Mehta, 2014). PKCβ is the PKC isoform most closely associated with the regulation of ROS activity in p66Shc (Ono et al., 1987),and it can be activated by both DAG and PTM. PKCβ activation inhibits autophagy and causes mitochondrial translocation in p66Shc (Figure 3) (Green et al., 2011). The binding of phosphorylated tyrosine to the SH2 domain of p66shc is mediated by another PKC isoform, PKCδ, which modulates the mitochondrial activity of p66shc (Morita et al., 2008).

##### 2.1.1.3 Oxidative stress in inflammatory states

Oxidative stress and inflammation exhibit a reciprocal relationship, with inflammation playing a pivotal role in the pathogenesis of AD (Wortmann et al., 2021; Zhu et al., 2023). Oxidative stress directly stimulates the infiltration of inflammatory cells, enhances the secretion of inflammatory cytokines, and promotes the production of inflammatory vesicles, thereby amplifying the inflammatory response. Cytokines secreted by inflammatory cells, in turn, induce oxidative stress, and excessive ROS further increase cytokine secretion (Lian et al., 2019).

###### 2.1.1.3.1 Neutrophils and oxidative stress

Oxidative stress is a crucial mediator of AngII receptor signaling in human neutrophils (El Bekay et al., 2003). Following attachment to the vascular endothelium, neutrophils migrate to the site of tissue damage, where they become activated and secrete various vasoactive substances as part of the inflammatory response. Once activated, neutrophils initiate intracellular signaling and oxidative bursts through interactions with specific receptors on the cell membrane (McPhail et al., 1995). The generation of ROS in neutrophils primarily results from the activation of NOX located on the plasma membrane. In the AD model induced by AngII, AngII significantly enhanced neutrophil and NOX activity by activating the AT1 receptor, leading to the production of large amounts of ROS. These ROS not only exacerbate oxidative stress but also promote monocyte adhesion to vascular endothelial cells and trigger the formation of extracellular traps (NETs) (Chrysanthopoulou et al., 2021). NET formation is accompanied by neutrophil death, a unique process distinct from apoptosis and necrosis, called NETosis. Ang II-induced NETosis is closely linked to ROS, and this process may influence arterial remodeling in aneurysms through two primary pathways. First, by degrading elastin, predominantly through MMPs. Neutrophils secrete and activate MMPs, such as MMP-8 and MMP-9, which accelerate the breakdown of the extracellular matrix. Excessive MMP expression compromises elastic and collagen fibers, rendering the aortic medial layer more fragile and increasing the likelihood of dissection. Second, NETosis promotes collagen accumulation, a process involving tissue factors (Chrysanthopoulou et al., 2021).

###### 2.1.1.3.2 Macrophages and oxidative stress

In mouse models of AD, the infiltration of immune cells-specifically T lymphocytes, neutrophils, and, most notably, macrophages-into the aorta marks the early stages of AD pathogenesis. Macrophages play a central role in driving inflammation within the aortic wall. As AD progresses, AngII promotes the migration of macrophages from the aortic adventitia into the media, a process facilitated by pathogenic mechanisms involving serum lipids (Tanaka et al., 2018; Li et al., 2020). Upon classical activation into the M1 phenotype, macrophages release a variety of chemokines and potent oxidants that contribute to tissue damage. Elevated levels of ROS further drive the polarization of macrophages toward the M1 phenotype, enhancing the release of inflammatory cytokines and intensifying the pro-inflammatory response (Liu et al., 2022). Macrophages also secrete MMPs, which play a critical role in the development of AD. In this context, the expression of MMPs such as MMP-8, MMP-9, and MMP-12 is significantly elevated, underscoring the importance of maintaining a balance between MMPs and their inhibitors, tissue inhibitors of metalloproteinases. An imbalance between these two groups can lead to the degradation of the extracellular matrix and subsequent arterial wall remodeling in AD (Cifani et al., 2015). In addition, interleukins (IL), a class of pro-inflammatory cytokines secreted by macrophages, play a significant role in the inflammatory process in AD (Shimizu et al., 2006). Notably, increased concentrations of IL-6, IL-8, IL-11, IL-12, IL-16, and IL-18 have been detected in the serum of individuals with AD (Figure 2). Vascular endothelial growth factor (VEGF), present in pro-inflammatory macrophages and ECs that compose the neovascular wall, promotes neoangiogenesis, further exacerbating inflammation and matrix degradation (Reinders et al., 2003; Del Porto et al., 2014). The Socs3 gene in macrophages may also have a protective role in the aorta during AD. Macrophage Socs3 deficiency leads to increased macrophage proliferation and inflammation, driving macrophages toward a tissue-destructive phenotype. This deficiency also disrupts the differentiation of VSMCs, while alleviating excessive inflammation and initiating tissue repair processes, including the proper regulation of VSMC function (Ohno-Urabe et al., 2018).

##### 2.1.1.4 Oxidative stress in aging

Aging is associated with decreased efficiency of mitochondrial respiration, leading to excessive ROS production (Kim et al., 2025). Dysfunctional mitochondria also fail to maintain cellular energy demands, exacerbating oxidative damage in VSMCs and ECs(Yardeni et al., 2025). Aging promotes a pro-inflammatory state, often termed “inflammaging,” which amplifies ROS generation (Balamurugan et al., 2024). This state is driven by increased production of pro-inflammatory cytokines, activation of the NF-κB pathway, and immune cell infiltration (Cho and Park, 2024). The resulting oxidative stress contributes to endothelial dysfunction and extracellular matrix (ECM) degradation, pivotal events in the development of AD (Ganizada et al., 2024).

Aging disrupts iron homeostasis, leading to intracellular iron accumulation, which promotes Fenton reactions that generate harmful hydroxyl radicals (Lee et al., 2024). This accumulation sensitizes cells to ferroptosis, a type of iron-dependent cell death, further weakening the aortic wall (Zhang H. et al., 2024). With age, the expression and activity of key antioxidant enzymes, such as SOD, GPX, and catalase, decline. This reduction limits the ability to neutralize ROS, increasing oxidative damage to cellular components (Du et al., 2024). The aging-associated increase in oxidative stress accelerates cellular senescence, VSMC apoptosis, and ECM degradation, all of which compromise the structural integrity of the aortic wall (Gao et al., 2023). Additionally, age-related endothelial dysfunction heightens vascular permeability and inflammatory responses, further predisposing the aorta to dissection under hemodynamic stress (Meng et al., 2021).

Targeting aging-related oxidative stress may provide novel therapeutic avenues for preventing and treating AD. Strategies could include enhancing mitochondrial function, restoring antioxidant capacity, and modulating iron metabolism to mitigate the deleterious effects of aging on vascular health.

### 2.2 Oxidative stress and ferroptosis

The accumulation of intracellular lipid peroxides and the heightened oxidative stress resulting from the Fenton reaction characterize ferroptosis as a distinct form of cell death. It is marked by specific morphological changes, including the depletion or absence of mitochondrial cristae, the breakdown of the mitochondrial outer membrane, and the condensation of the mitochondrial membrane (Cao and Dixon, 2016; Yu et al., 2017). Oxidative stress, along with the loss of selective permeability of the plasma membrane, arises from lipid peroxidation of membrane components, contributing to these cellular abnormalities (Xie et al., 2016). The generation of ROS in ferroptosis involves multiple sources. In addition to ROS produced by iron-catalyzed Fenton reactions, NADPH-dependent lipid peroxidation and GSH depletion play pivotal roles in the initiation of ferroptosis (Dixon et al., 2012).

Iron is primarily found in organisms as trivalent iron ions (Fe³⁺), which bind tightly to transferrin to form a circulating iron complex (Kwon et al., 2015). This complex is internalized into the cell via transferrin receptor 1 (TFR1) on the cell membrane and is subsequently transported to the endosomal compartments. Within the endosome, Fe³⁺ undergoes reduction to divalent iron ions (Fe^2^⁺), catalyzed by the enzyme six-transmembrane epithelial antigen of prostate 3(STEAP3 (with ferric reductase activity). The generated Fe^2^⁺ is then released from the endosome into the cytoplasmic iron pool, which remains in an unstable state, mediated by the divalent metal transporter 1 (DMT1), also known as SLC11A2. When intracellular iron levels exceed the cellular demand, excess iron is stored in ferritin. Iron efflux from the cell relies on the membrane protein iron transport protein (also known as SLC11A3), an iron efflux pump, which re-oxidizes intracellular Fe^2^⁺ to Fe³⁺ and facilitates its transmembrane translocation out of the cell. This process helps maintain a dynamic balance of intracellular iron levels.

#### 2.2.1 Sources of oxidative stress in ferroptosis

Ferroptosis occurs in two distinct phases. The first phase involves an overload of intracellular iron, which drives the generation of substantial quantities of ROS through Fenton reactions. The second phase is marked by a disruption of the intracellular antioxidant system, impairing the cell’s ability to effectively reduce excessive phospholipid hydroperoxides. This imbalance undermines the structural integrity of the cell membrane and disrupts mitochondrial function, ultimately leading to cellular ferroptosis.

##### 2.2.1.1 Intracellular iron overload

The Fenton reaction produces hydroxyl radicals (·OH) and hydroperoxides through the interaction of Fe^2^⁺ with H₂O₂ (Toyokuni, 2002). In this reaction, Fe^2^⁺ is oxidized to Fe³⁺, while H₂O₂ is reduced, leading to the formation of highly reactive hydroxyl radicals. These radicals are exceptionally potent oxidants, targeting PUFAs in cell membranes and causing lipid peroxidation. Furthermore, Fe³⁺ can be reduced back to Fe^2^⁺, enabling it to participate repeatedly in the Fenton reaction. This process results in continuous intracellular ROS generation, creating a positive feedback loop that accelerates lipid peroxidation and ferroptosis. In addition to the free radicals generated by the Fenton reaction, lipoxygenases (LOXs) are key enzymes that drive lipid peroxidation. LOXs can directly oxidize PUFAs in cell membranes, producing lipid peroxides that further exacerbate ferroptosis progression (Kagan et al., 2017). A critical factor in ferroptosis is the oxidation of PUFAs and phosphatidylethanolamine (PE), which leads to the formation of lipid hydroperoxides (LOOH). This oxidative process triggers signaling pathways that propagate and drive ferroptosis (Yang and Stockwell, 2016). The enzyme ACSL4 (Acyl-CoA Synthetase Long-Chain Family Member 4), part of the long-chain acyl-CoA synthetase family, catalyzes the synthesis of arachidonoyl-CoA (AA-CoA) from arachidonic acid (AA). Under the action of lysophosphatidylcholine acyltransferase 3 (LPCAT3), AA-CoA is incorporated into PE to form PE-AA. This PE-AA is then oxidized by LOX, generating lipid peroxides that induce ferroptosis. Inhibition of both ACSL4 and LPCAT3 reduces cellular lipid peroxide production, thereby effectively inhibiting ferroptosis (Kagan et al., 2017).

##### 2.2.1.2 Regulation of the antioxidant system

The antioxidant system that mitigates ferroptosis consists of multiple components working together to maintain cellular redox balance, inhibit lipid peroxidation, and prevent the onset and progression of ferroptosis. Disruption at any stage of this system can lead to an imbalance, promoting ferroptosis.

GPX4 plays a critical role in regulating ferroptosis by inhibiting lipid peroxidation and preventing the activation of arachidonic acid (AA) metabolic enzymes, thus providing protective effects against ferroptosis (Yang et al., 2014). Another key component of the antioxidant defense system is the xc⁻ system, a transmembrane amino acid antiporter embedded within the phospholipid bilayer. It is composed of two subunits, SLC7A11 and SLC3A2, which work together to facilitate the exchange of cysteine and glutamate across the cellular membrane, contributing to the maintenance of redox homeostasis (Dixon et al., 2012).

Several novel regulatory mechanisms, distinct from the conventional GPX4/GSH-dependent ferroptosis pathway, have been identified. These include the FSP1-CoQ10 pathway, the membrane repair pathway mediated by the endosomal sorting complex required for transport III (ESCRT-III), and the pathway involving guanosine triphosphate cyclohydrolase one and tetrahydrobiopterin (GCH1-BH4) (Dai et al., 2020; Kraft et al., 2020; Zeng et al., 2022). As a crucial endogenous lipophilic antioxidant, coenzyme Q10(CoQ10) acts as a reversible redox carrier in electron transfer processes within both the plasma and Golgi membranes. It effectively scavenges lipid peroxide radicals, thereby inhibiting ferroptosis (Ernster and Forsmark-Andrée, 1993; Farquhar and Palade, 1998). CoQ10 is the primary target of the FSP1 pathway, where FSP1 catalyzes the reduction of non-mitochondrial CoQ10 using NADPH (Morré and Morré, 2011). The ESCRT-III complex, a key member of the ESCRT family of complexes, plays a vital role in regulating various cellular processes. Standard inducers of ferroptosis, such as erastin and RSL3, promote the aggregation of ESCRT-III subunits, and knockout of ESCRT-III exacerbates ferroptosis (Dai et al., 2020). BH4 is a potent radical-scavenging antioxidant that prevents ferroptosis by reducing lipid peroxidation (Soula et al., 2020). In the biosynthesis of BH4, GCH1 functions as the rate-limiting enzyme. A genome-wide activation screening identified GCH1 as the most critical gene involved in ferroptosis prevention, acting independently of the GPX4/GSH axis (Kraft et al., 2020).

##### 2.2.1.3 Dysregulation of the antioxidant system

When the antioxidant system that regulates ferroptosis becomes disrupted, cells lose the ability to eliminate lipid peroxides and ROS, leading to membrane damage and the onset of ferroptosis. Several factors can contribute to this disruption, including GSH depletion, GPX4 inhibition, malfunction of the xc⁻ system, impaired ferritin storage, CoQ10 deficiency, or disruption of Nrf2 signaling. Each of these factors accelerates the process of ferroptosis. A diet high in iron can induce ferroptosis in mice, and adding iron to the extracellular matrix increases cellular sensitivity to ferroptosis (Wang et al., 2017). In iron-sensitive cells, transferrin levels rise, while the expression of the iron transporter protein ferroportin decreases (Gao et al., 2015). Furthermore, nuclear receptor coactivator 4 (NCOA4)-mediated phagocytosis of ferritin facilitates the release of iron, thereby intensifying ferroptosis (Mancias et al., 2014). Small molecules such as erastin, RSL3, FIN56, and FINO2 can also induce ferroptosis by inhibiting GPX4 enzyme activity (Dolma et al., 2003; Gaschler et al., 2018). Since GPX4 requires GSH as a cofactor, inhibiting GSH biosynthesis is another means of inducing ferroptosis. Cystine is essential for intracellular GSH synthesis, so inhibiting the xc⁻ system, which facilitates cystine uptake, impairs GSH synthesis. This results in decreased intracellular antioxidant capacity, the accumulation of lipid ROS, and ultimately, oxidative damage leading to ferroptosis (Dixon et al., 2014). Inhibition of GPX4 specifically leads to the accumulation of lipid peroxides (Brigelius-Flohé and Maiorino, 2013),making cells with reduced GPX4 levels more susceptible to ferroptosis (Yang and Stockwell, 2008). The activating transcription factor family, including ATF3 and ATF4, is linked to the function of the xc⁻ system (Gaschler et al., 2018). When intracellular redox balance is disturbed, ATF3 mRNA levels increase, and the activated ATF3 tightly binds to the promoter of SLC7A11, suppressing its expression and promoting iron-dependent cell death under oxidative stress conditions. P53, a well-known tumor suppressor gene, can mitigate lethal ROS accumulation at low to moderate levels of ROS. However, under conditions of high ROS levels, P53 induces ferroptosis by inhibiting the transcription of SLC7A11.

##### 2.2.1.4 Oxidative stress mediated by endoplasmic reticulum (ER) dysfunction

The endoplasmic reticulum (ER) is a critical organelle involved in protein folding, calcium homeostasis, and lipid metabolism (Larrea et al., 2025). Under pathological conditions such as aortic dissection (AD), ER dysfunction results in the accumulation of misfolded proteins and disruption of calcium signaling, leading to ER stress (Chiarelli et al., 2024). This stress can initiate the unfolded protein response (UPR), which, when sustained, exacerbates oxidative stress and contributes to cell death. ER and mitochondria are physically and functionally connected through mitochondria-associated membranes (MAMs) (Chen et al., 2024). These contact sites play a pivotal role in ROS production and lipid metabolism, both of which are integral to ferroptosis. Dysregulated MAM signaling has been shown to amplify oxidative stress and disrupt redox balance, potentially aggravating vascular smooth muscle cell (VSMC) dysfunction and endothelial injury in AD (Liu et al., 2024b). As highlighted in recent studies, the crosstalk between ER and mitochondria is a key contributor to ROS-mediated damage, emphasizing the significance of MAMs in ferroptosis pathways (Shi et al., 2022).

Furthermore, excessive ER stress activates UPR pathways, such as PERK-eIF2α-ATF4, which can exacerbate oxidative damage by increasing ROS levels and depleting cellular antioxidant defenses (Lu et al., 2024). This mechanism has been implicated in the pathogenesis of various vascular diseases, including AD. Recent findings have further clarified the role of ER stress in triggering inflammatory responses and ferroptosis, highlighting its potential as a therapeutic target (Qi Z. et al., 2024). Therapeutic strategies aimed at modulating ER stress have shown promise in reducing oxidative damage and preventing ferroptosis. For instance, chemical chaperones, such as 4-phenylbutyric acid, and inhibitors of UPR pathways have demonstrated efficacy in preclinical models by alleviating ER stress and restoring redox homeostasis (Zha et al., 2024).

## 3 The role and mechanism of ferroptosis in AD

AD is a severe vascular disease characterized by the degeneration of the aortic media (Jiang et al., 2016). Emerging evidence suggests that programmed cell death plays a critical role in the depletion of VSMCs and the progression of AD (Li R. et al., 2018; Luo et al., 2020). Among these cell death pathways, ferroptosis has been implicated in aortic degeneration and the formation of AD. During the progression of AD, ferroptosis becomes activated, and its inhibition by liproxstatin-1 has been shown to partially alleviate aortic degeneration and slow the progression of AD in mouse models (Li et al., 2022). In patients with thoracic aortic aneurysm and dissection (TAAD), there is a notable downregulation of key ferroptosis regulators, including SLC7A11 and FSP1, while the RNA methyltransferase METTL3 is significantly upregulated (Li et al., 2022). These findings suggest a dysregulation of ferroptosis-related pathways in the pathogenesis of TAAD.

### 3.1 Ferroptosis and VSMCs

The death of VSMCs is a primary contributor to medial degeneration in the aorta (Guo et al., 2019). This degeneration of the medial layer is a histopathological feature common to both ascending thoracic aortic aneurysm (ATAA) and acute Stanford type A AD (ATAAD), classifying them as two subtypes of thoracic aortic diseases. However, unlike ATAA, ATAAD is characterized by intimal rupture and the rapid formation of a false lumen. The damage and loss of VSMCs likely play a pivotal role in shaping the microenvironment surrounding the false lumen (Isselbacher, 2005).

A substantial number of hemoglobin-rich red blood cells are present within the thrombus of the false lumen in ATAAD. HO-1, the primary isoform of heme oxygenase, is highly induced by hypoxia, oxidative stress, and various internal environmental changes. As a key enzyme in heme catabolism, HO-1 breaks down heme, releasing iron, which can ultimately trigger ferroptosis (Imuta et al., 2007). When blood enters the false lumen in ATAAD, hypoxia-inducible factor 1-alpha (HIF-1α) stimulates the robust expression of HO-1 in VSMCs in response to oxidative stress. This leads to the degradation of heme by HO-1, releasing significant amounts of free iron, which initiates ferroptosis in VSMCs. As VSMCs undergo ferroptosis, HO-1 may be released into the microenvironment of the false lumen, perpetuating a vicious cycle of iron accumulation and ferroptosis. This positive feedback loop, involving oxidative stress, HO-1 upregulation, and ferroptosis, is thought to contribute to the expansion of the false lumen and the progression of AD (Song et al., 2023). The unique microenvironment within the false lumen exacerbates ferroptosis, which is further induced by oxidative stress. This creates a positive feedback loop that fuels inflammatory and immune responses, accelerating disease progression (Yu et al., 2021; Chen et al., 2023).

Following vascular injury, VSMCs undergo a phenotypic transformation, shifting from a dormant contractile state to an activated synthetic state, relocating from the media layer to the intima. In this activated state, VSMCs proliferate rapidly and synthesize and secrete extracellular matrix components, leading to neointima formation and vascular stenosis (Jackson, 1994). The carotid artery ligation model has demonstrated that ferroptosis plays a critical role in neointima formation, as it promotes neointimal proliferation, while its inhibition reduces this proliferation (Zhang et al., 2022). Cigarette smoke extract induces ferroptosis in VSMCs, a process that cannot be reversed by apoptosis or necroptosis inhibitors but can be mitigated by ferrostatin-1 (Fer-1), liproxstatin-1, and iron chelators (Sampilvanjil et al., 2020). ROS act as regulatory factors in the phenotypic transformation of VSMCs. They promote VSMC dedifferentiation, proliferation, and migration through the NF-κB/mTOR/P70S6K signaling pathway (Griendling and Ushio-Fukai, 1998; Clempus and Griendling, 2006; Lu et al., 2018). FSP1, originally identified as a flavoprotein regulating apoptosis, has recently been shown to inhibit ferroptosis by catalyzing the reconstitution of CoQ10. This process reduces lipid peroxidation by utilizing NAD(P)H (Bersuker et al., 2019; Doll et al., 2019).

### 3.2 Ferroptosis and vascular endothelial cells

The inner surface of blood vessels is lined by a highly dynamic monolayer of ECs known as the endothelium. This layer is essential for numerous vascular functions, including maintaining vascular tone, balancing coagulation and anticoagulation, modulating immune responses, and regulating the proliferation and migration of VSMCs (Förstermann et al., 2017; Godo and Shimokawa, 2017).

ROS in ECs primarily originate from mitochondria, NOX, endothelial nitric oxide synthase (eNOS) decoupling, and xanthine oxidase (XO) (El Assar et al., 2013; Incalza et al., 2018). Under normal conditions, ROS play essential roles in physiological processes, such as host defense, post-translational modification of proteins, cellular signaling pathways, gene expression regulation, and cell differentiation, all of which are critical for maintaining cellular homeostasis (Bedard and Krause, 2007). However, excessive ROS production can lead to significant damage to ECs, resulting in endothelial dysfunction and, in some cases, cell death (El Assar et al., 2013). During ROS-induced endothelial dysfunction, ECs upregulate the expression of pro-inflammatory cytokines, including interleukin-1β (IL-1β) and interleukin-18 (IL-18). They also increase the expression of adhesion molecules such as intercellular adhesion molecule-1 (ICAM-1) and vascular cell adhesion molecule-1 (VCAM-1). These changes are closely linked to the inflammatory response, further exacerbating endothelial damage (Chen et al., 2017).

### 3.3 Interventions for ferroptosis in AD

Ferroptosis, a recently discovered form of programmed cell death, is closely associated with the pathological progression of AD. Regulating ferroptosis-related molecules and pathways presents a promising approach for developing new therapeutic interventions for AD. Treatment strategies targeting AD through the modulation of ferroptosis primarily focus on regulating iron metabolism, enhancing antioxidant defense systems, inhibiting lipid peroxidation, and strengthening cellular protective mechanisms.

Pharmacological interventions targeting ferroptosis hold promise for the treatment and management of AD (Table 1). For instance, liproxstatin-1, a potent inhibitor of lipid peroxidation, has demonstrated efficacy in preclinical studies by mitigating VSMC ferroptosis and preserving aortic wall integrity (Li et al., 2022). Similarly, iron chelators like deferoxamine effectively reduce intracellular iron accumulation, which is a critical driver of ferroptosis, thus protecting against oxidative stress-induced damage in the aortic wall (Table 1) (Sampilvanjil et al., 2020). Activation of the NRF2 pathway by agents such as bardoxolone methyl enhances the antioxidant defense system, providing a potential strategy to counteract chronic oxidative stress (Graber et al., 2022). These interventions could complement existing therapeutic approaches, addressing the underlying mechanisms of aortic wall degradation and reducing disease progression. However, further research is needed to validate their efficacy and safety in clinical settings.

**TABLE 1:** Ferroptosis mechanisms and therapeutic strategies in aortic dissection.

Category	Target	Role	Therapeutic strategy	References
Regulation of iron metabolism	TFR1	Mediates cellular iron uptake	Inhibit TFR1 activity to reduce iron overload	Li et al. (2021)
	DMT1	Releases iron from endosomes to cytoplasm	Suppress DMT1 to limit free iron levels	Xie et al. (2024)
	Ferritin	Stores intracellular excess iron	Enhance ferritin to balance iron homeostasis	He et al. (2024b)
	Iron chelators	Prevents iron-driven ROS generation	Apply iron chelators to reduce oxidative stress	Sampilvanjil et al. (2020)
Antioxidant systems	GPX4	Inhibits lipid peroxidation	Activate GPX4 to counter ferroptosis	Wang et al. (2023)
	NRF2	Regulates antioxidant and iron metabolism	Boost NRF2 signaling to enhance cell defenses	Liu et al. (2024a)
	System xCT	Supports GSH synthesis for redox balance	Stimulate system xCT or supply GSH.	Qi et al. (2024a)
Lipid metabolism regulation	ACSL4	Drives lipid peroxidation processes	Inhibit ACSL4 to limit lipid peroxidation	Qi et al. (2024b)
	LPCAT3	Contributes to phospholipid oxidation	Target LPCAT3 to reduce oxidative damage	Zhang et al. (2024b)
	CoQ10	Neutralizes lipid peroxide radicals	Increase CoQ10 or activate FSP1 for protection	Fan et al. (2024)
ROS pathways	NOX	Generates ROS, intensifying oxidative stress	Inhibit NADPH oxidase to lower ROS levels	Chen et al. (2021)
	HO-1	Regulates iron and oxidative stress	Modulate HO-1 to mitigate vascular damage	Song et al. (2023)
Direct ferroptosis inhibitors	Liproxstatin-1	Blocks lipid peroxidation cascade	Use Liproxstatin-1 to slow AD progression	Li et al. (2022)
	Ferrostatins	Prevent ferroptosis-related cell damage	Apply Ferrostatins for targeted interventions	Shi et al. (2023)

#### 3.3.1 Targets for iron metabolism regulation

Iron metabolism imbalance is a central mechanism driving ferroptosis, and modulating iron metabolism can effectively reduce vascular cell damage induced by this process.

Iron chelators bind to excess intracellular free iron, preventing iron-catalyzed Fenton reactions from generating excessive ROS, thereby mitigating oxidative stress in VSMCs and ECs. The use of selective iron chelators is a promising therapeutic strategy for removing excess iron and protecting against ferroptosis-related damage (Chen et al., 2020).

Targeted regulation of proteins involved in iron homeostasis, such as TFR1 and DMT1, can reduce the uptake of intracellular free iron, thereby decreasing the occurrence of ferroptosis. TFR1 facilitates the transfer of iron from the extracellular environment into cells, thereby increasing intracellular iron reserves, which are essential for ferroptosis induction. In this context, TFR1 plays a pivotal role in iron transport, ensuring an adequate supply of iron within the cell to support ferroptosis (Yang and Stockwell, 2008).

#### 3.3.2 Targets of the antioxidant system

The process of ferroptosis is accelerated when the antioxidant system is imbalanced, whereas enhancing antioxidant capacity can effectively inhibit ferroptosis within the aortic wall. Specifically, an imbalance in the antioxidant system promotes the progression of ferroptosis, while bolstering antioxidant defenses acts as a regulatory mechanism, slowing ferroptosis in the aortic tissue.

##### 3.3.2.1 Antioxidant mechanisms mediated by GPX4

The conversion of lipid hydroperoxides into non-toxic lipid alcohols is a key function of GPX4, which plays a critical regulatory role in ferroptosis by effectively inhibiting lipid peroxidation (Xie et al., 2023). The XC⁻/GSH/GPX4 axis is a fundamental component of the antioxidant defense system, playing an essential role in preventing ferroptosis mediated by lipid peroxidation. Together, GPX4 and the XC⁻/GSH/GPX4 axis maintain a balance that mitigates the harmful effects of lipid peroxidation in ferroptosis. Nrf2 directly influences GPX4 and also modulates its activity indirectly through the production of GSH. FSP1 and GPX4 represent two primary, parallel defense mechanisms against ferroptosis. Inhibition of FSP1 leads to enhanced ferroptosis, with FSP1’s protective effect against ferroptosis being mediated by CoQ10. CoQ10 captures lipid peroxide radicals, while FSP1 utilizes NAD(P)H to catalyze the regeneration of CoQ10 (Doll et al., 2019).

##### 3.3.2.2 Antioxidant mechanisms mediated by NRF2

The transcription factor NRF2 plays a crucial role in regulating cellular antioxidant responses by orchestrating the expression of genes that counteract oxidative and electrophilic stress. Sensitivity to ferroptosis is directly linked to NRF2 levels: upregulation of NRF2 can protect cells from ferroptosis, while its downregulation increases the susceptibility of cancer cells to ferroptosis-inducing agents (Sun et al., 2016; Fan et al., 2017). In addition to its role in oxidative stress defense, NRF2 is central to mediating iron and heme metabolism, by targeting key proteins involved in these processes (Kerins and Ooi, 2018). Among NRF2’s targets are the ferritin light (FTL) and heavy (FTH1) chains, which are essential for iron storage, and the iron export protein SLC40A1, which facilitates the export of iron from cells (Harada et al., 2011; Agyeman et al., 2012). NRF2 also regulates cellular antioxidants, iron metabolism, and metabolic intermediates. Notably, it controls two critical targets involved in ferroptosis: xCT and GPX4 (Dodson et al., 2019). By modulating these targets, NRF2 can mitigate the risk of ferroptosis. Given its pivotal role in regulating oxidative stress and iron homeostasis, targeting NRF2 presents a promising therapeutic strategy for treating diseases characterized by lipid peroxidation and ferroptosis.

##### 3.3.2.3 Antioxidant mechanisms mediated by cystine/glutamate exchange system (system xc⁻)

The system xc⁻, a sodium-dependent antiporter, consists of two subunits: 4F2hc (SLC3A2) and xCT (SLC7A11). This system mediates the 1:1 exchange of cystine from the extracellular space with intracellular glutamate. Additionally, system xc⁻ facilitates the conversion of cystine into cysteine, a critical precursor required for the biosynthesis of GSH, an essential antioxidant that helps maintain cellular redox balance (Sato et al., 1999; Conrad and Sato, 2012). GSH plays a vital role in restoring intracellular redox equilibrium, particularly after the production of ROS. Erastin, a known inhibitor of system xc⁻, impedes cystine uptake and, consequently, disrupts cysteine-dependent GSH synthesis. This blockage leads to a lethal accumulation of cytoplasmic and lipid ROS, thereby triggering ferroptosis (Dixon et al., 2012). Mechanistically, the inhibition of system xc⁻ by compounds such as erastin, SAS, or sorafenib results in significant depletion of intracellular GSH. This depletion severely disrupts the cell’s redox balance, leading to an excessive accumulation of ROS. The ensuing oxidative stress drives iron-dependent cell death, known as ferroptosis, which is characterized by the toxic accumulation of ROS to harmful levels (Ishii et al., 1981; Dixon et al., 2014).

##### 3.3.2.4 Antioxidant mechanisms mediated by long-chain acyl-CoA synthetase (ACSLs)

The ACSLs family of enzymes plays a crucial role in lipid metabolism. Microarray analyses, conducted using a CRISPR-based genome-wide screening system in ferroptosis-resistant cell lines, revealed a significant downregulation of ACSL4 in these cell lines. ACSL4 is essential for the induction of ferroptosis, primarily through the accumulation of oxidized phospholipids (PL) in the cell membrane (Doll et al., 2017; Li and Li, 2020). ACSL4 knockout (KO) cells exhibit resistance to RSL3, a GPX4 inhibitor and ferroptosis inducer. Conversely, overexpression of ACSL4 in ferroptosis-resistant cell lines, such as LNCap prostate cancer and K562 chronic myeloid leukemia lymphoblasts, markedly enhances their sensitivity to ferroptosis (Yuan et al., 2016; Doll et al., 2017). ACSL4 not only serves as a sensitive marker for ferroptosis but also acts as a critical regulator of lipid metabolism in this process. The ACSL4 gene encodes an enzyme that catalyzes the conversion of arachidonic acid (AA) and adrenaline (ADA) into their activated forms, AA-CoA and ADA-CoA, respectively. This biochemical process triggers the formation of lipid peroxides (LPOs), which are pivotal in driving ferroptosis. The accumulation of LPOs results in oxidative damage to cell membranes, contributing to the progression of iron-dependent cell death. The lipid peroxidation induced by ACSL4 is both fundamental and indispensable for the execution of ferroptosis. However, its role as a potential therapeutic target for ferroptosis in conditions like AD remains to be explored in future research.

## 4 Outlook and future perspectives

AD is a life-threatening vascular condition characterized by complex pathological processes in which oxidative stress, cell death, and inflammation play pivotal roles. Ferroptosis, a form of iron-dependent cell death, is closely associated with excessive iron accumulation, lipid peroxidation, and imbalances in antioxidant defenses, particularly within VSMCs and ECs. Ferroptosis not only accelerates the phenotypic transformation of VSMCs and the formation of neointima but also induces endothelial cell damage, compromising vascular integrity and facilitating the development and progression of AD.

Regulating ferroptosis-related molecules and pathways—such as inhibiting lipid peroxidation, enhancing antioxidant systems, and modulating intracellular iron homeostasis—offers a promising approach to mitigating ferroptosis-induced vascular damage. Consequently, targeting these ferroptosis-related pathways holds significant potential as a novel therapeutic strategy for treating AD.

Despite early investigations into the role of ferroptosis in AD, the underlying mechanisms and potential therapeutic targets require further exploration. Future research should focus on elucidating the mechanistic role of ferroptosis in AD, particularly by examining the regulatory networks governing ferroptosis in VSMCs and ECs, and understanding the interplay between oxidative stress, lipid metabolism, and iron homeostasis. Additionally, identifying molecular biomarkers associated with ferroptosis could aid in the early diagnosis and prognostic evaluation of AD, facilitating the timely identification of high-risk patients for intervention.

Further studies should also investigate additional regulatory molecules involved in ferroptosis, such as GPX4, ACSL4, and FSP1, and develop specific drugs targeting these pathways. Potential therapeutic approaches may include Antioxidant Enhancers, antioxidants, and inhibitors of lipid peroxidation. A deeper understanding of ferroptosis mechanisms will enable the clinical application of targeted therapies, allowing for the assessment of their safety and efficacy in patients with AD, as well as the development of personalized treatment plans.

The prospects for ferroptosis research in AD are vast. By leveraging targeted intervention strategies, there is potential to provide new therapeutic options for patients, ultimately reducing the incidence and mortality associated with this condition.
